# Randomized controlled trials vs. observational studies: why not just live together?

**DOI:** 10.1186/s12871-016-0265-3

**Published:** 2016-10-21

**Authors:** David Faraoni, Simon Thomas Schaefer

**Affiliations:** 1 Department of Anesthesia and Pain Medicine, The Hospital for Sick Children, University of Toronto, Toronto, Canada; 2 Department of Anesthesiology, Ludwig-Maximilians University of Munich, Marchioninistraße 15, 81377 München, Germany

**Keywords:** Randomized controlled trials, Observational studies, Study planning, Metaanalysis, Good clinical practice

## Abstract

Randomized controlled trials (RCTs) are considered the gold standard for clinical research, thus having a high impact on clinical guidelines and our daily patients’ care. However, various treatment strategies which we consider “evidence based” have never been subject to a prospective RCT, as we would rate it unethical to withheld an established treatment to individuals in an placebo controlled trial.

In a recent BMC Anesthesiology publication, Trentino et al. analyzed the usefulness of observational studies in assessing benefit and risk of different transfusion strategies. The authors nicely reviewed and summarized similarities and differences, advantages and limitations, between different study types frequently used in transfusion medicine. In this interesting article, the authors conclude, that ‘when comparing the results of observational studies with RCTs assessing transfusion outcomes, it is important that one consider not only the study method, but also the key elements of the study design’. Thus, in this commentary we now discuss the pro’s and con’s of different study types, even irrespective of transfusion medicine.

## Background

Over the past decades, requirements for the design of clinical studies increased, favouring randomized, controlled trials (RCTs). In this context, benefit and risk associated with allogeneic blood product transfusions have been discussed and debated in a large number of publications. If only a few prospective RCTs compared a liberal with a restrictive transfusion strategy in different medical and surgical populations, a large number of retrospective observational studies have been published leading to sometimes conflicting results [1, 2]. In a recent *BMC Anesthesiology* publication, Trentino et al. addressed an important question: “Should we ignore the results obtained from observational studies when assessing the benefit and the risk of different transfusion strategies [3]?”. The authors nicely reviewed and summarized the similarities and differences, advantages and limitations, between different study designs frequently used in transfusion medicine. The authors concluded that “when comparing the results of observational studies with RCTs assessing transfusion outcomes, it is important that one consider not only the study method, but also the key elements of the study design”.

## Main text

With the increasing importance of evidence based medicine, RCTs are now typically regarded as the “gold standard” to evaluate the efficacy of a therapy or an intervention intended to improve outcome. Some consider RCTs to be the only valid design to evaluate therapeutic efficacy. The strengths of RCTs are obvious and include the development of a prospective study protocol with strict inclusion and exclusion criteria, a well-defined intervention, and predefined endpoints [4]. All of those being usually absent or defined ‘a posteriori’ in observational studies, which makes the interpretation of the results difficult. However, our daily clinical practice is mainly based on the understanding of the pathophysiology, and how any given interventions may influence that pathophysiology to improve outcomes [5]. In addition, clinical decision making is still based on behaviours and treatments which have never been evaluated in clinical trials, considering that some interventions may never be subject to a randomization. As an example, it is obvious that a RCT to assess the influence of intraoperative opioids on sympathetic nervous system activation and surgical pain compared to a placebo would be considered highly unethical, and will never be performed. Thus, observational trials are sometimes the only option to get data on specific scientific questions.

Transfusion medicine is a good example of how pathophysiology can influence the effectiveness or safety of a treatment when applied in different clinical circumstances or populations. Among various studies that compared the effect of two transfusion triggers on outcomes in different populations, some of them indicated that a restrictive transfusion strategy (transfusion threshold Hb > 7–8 g/dL) was at least as good as a liberal transfusion strategy (transfusion threshold Hb > 9–10 g/dL) [6, 7], while other studies suggested that a restrictive transfusion strategy could be harmful when applied to other populations, like patients with neoplasm or coronary artery disease [8, 9]. That being said, the conflict in results between prospective studies doesn’t mean that we should reconsider the findings of those well-designed trials, but this should be considered as a good opportunity to highlight the limitations of large RCTs and the aspect that could not be assessed by a single study. The application of strict inclusion and exclusion criteria often lead to the inclusion of a very small proportion of the patients that we are used to deal with in our daily practice, which means that the studied population does not reflect the real world’. Furthermore, when analyzing RCT one needs to take into account the control group used. A recent meta-analysis showed that a randomized placebo-controlled design was more often used in studies funded by pharmaceutical companies, i.e. as shown for psoriasis [10]. To test the overall efficiency of a new drug placebo controls are appropriate, however comparing two different therapeutic options might be necessary to show that a new, usually more expansive, treatment is superior to the established gold standard. This is of particular importance, as new medications should show their additional benefit in comparison to existing therapeutic strategies and not compared to placebo, which at least in some countries, is the premise for being covered by health care insurances [11].

Although, RCTs are Masterpieces to assess the efficacy of a treatment in a specific population (e.g. can the treatment work under ideal circumstances?), alternatives are required to assess the effectiveness of the same therapy (e.g. will the treatment work in real-world circumstances) [12]. The progresses made in term of sophisticated statistical methods (e.g. multivariable logistic regression, propensity matched analysis) have pushed researchers to consider observational studies as an easily accessible and cheap method to look at the safety and effectiveness of different therapeutic strategies, without the need to spend a lot of money randomizing a large number of patients [13]. One might argue that large observational trials using propensity score matching and appropriate multivariate regression analyses might better reflect the ‘real clinical world’ than a RCT performed in a homogenous subgroup of patients. Most important, the quality of the results obtained from those studies is highly influenced by the quality of the data collected, the quality of the method applied to adjust for potential confounders as well as the selection of the confounders, and finally the quality of the interpretation of the results and limitations.

**Fig. 1:**
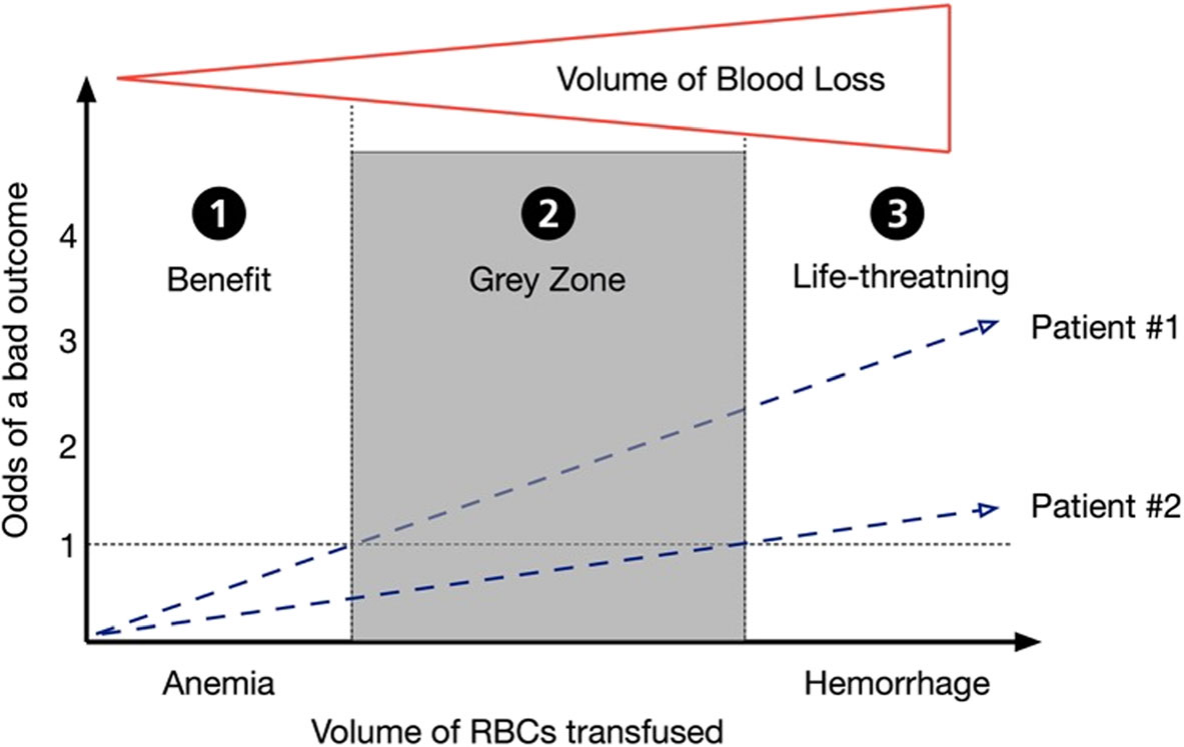
Relationship between the volume of red blood cells transfused and the odds of a bad outcome in two different patients, after taking into consideration the underlying condition (e.g. anemia [Zone 1], hemorrhage [Zone 3]) and the volume of blood loss

Although it would be desirable to rely solely on RCTs to guide clinical practice, it is simply impossible. As illustrated in Fig. 1, the relationship between transfusion and outcome is far more than a simple relationship between transfusion (yes/no) and outcome (yes/no). Not only the volume of blood products transfused influences the odds of a bad outcome [14], but the underlying condition leading to the transfusion (e.g. anemia and/or hemorrhage) is also a key player [15]. In addition, patient’s characteristics may or may not influence the tolerance to any of those conditions, and all of those aspects are crucial in the understanding of the relationship between transfusion and outcome. If treatment of anemia through the transfusion of small volume of RBCs (Zone 1) may be beneficial in some circumstances (e.g. when alternatives, like preoperative optimization with iron, are not available), or the transfusion could be life-saving in the context of life-threatening hemorrhage (Zone 3) [16], there is a grey zone in-between (Zone 2) where some patients will benefit from a transfusion while other will be harmed by equal amounts and types of transfusion. It is also obvious that no RCTs or observational studies will ever be able to dissociate the effect of massive bleeding and massive transfusion (Zone 3) on the odds of a bad outcome, since it is absolutely impossible to compare massively bleeding patients that got transfused with those who did not receive any transfusions. Considering that the spectrum of transfusion medicine is extremely large and complicated, and represents a large population of patients with different co-morbidities and characteristics, it is extremely challenging to address all the different clinical scenarios into a single RCT or observational study.

Apart from RCTs or observational studies even basic research is currently in the focus as data obtained i.e. in mice obviously cannot be translated one to one to humans and the clinical setting [17]. However, it is hard to believe that modern research would work without animal studies, as analyzing signaling pathways in various conditions or the investigation of new therapeutic approaches as well as the performance of life threatening study protocols can only be done using animals. However, especially basic research can highly benefit from assuring data obtained from animals by adding data from an observational trial in humans, i.e. to validate alteration, found following a defined intervention in mice, in our clinical patients [18]. Thus, translational research using observational studies allows us to find associations between basic research and clinical patients’, and this even long before new, experimental therapeutic strategies could be approved and tested in RCTs.

## Conclusions

Although it is important to understand the strengths and limitations of both RCTs (efficacy studies) and observational studies (effectiveness studies), none of the study designs should be considered in isolation since all types of evidence rely primarily on the rigour with which individual studies were conducted (regardless of the methodological approach) and the care with which they are interpreted [19]. Interpretation of the results obtained from both RCTs and observational studies can help understand the efficacy/effectiveness and safety of a therapeutic option. Meta-analyses using both RCT and observational studies should be used to highlight some questions that neither a RCT, nor an observational study would have the ability to solve by themselves. As suggested by Trentino et al. [3], the results obtained from both RCTs and observational studies should be interpreted knowing the characteristics of the population, including the control group used and the method used to assess the efficacy and the safety of the treatment, with a good understanding of the potential limitations and aspects that the study was not able to address. While dressing the general picture, both RCTs and observational studies should be included in our reflection, considering that each study design could bring an important piece of information in the interpretation of the safety, efficacy, and effectiveness of a therapeutic option in different populations. Regardless of the study type, it remains our accountability to scrutinize methods, controls and conclusions drawn in all the paper we read. Thus reviewing and discussing this, as done by Trentino et al. in their recent paper regarding transfusion medicine, is highly helpful.

## Abbreviation

RCT: Randomized controlled trials
